# Editorial: Experts' opinions on aging and public health

**DOI:** 10.3389/fpubh.2023.1322841

**Published:** 2024-01-04

**Authors:** Marcia G. Ory, Matthew Lee Smith

**Affiliations:** ^1^Center for Community Health and Aging, Texas A&M University, College Station, TX, United States; ^2^School of Public Health Department of Environmental and Occupational Health, Texas A&M University, College Station, TX, United States; ^3^School of Public Health Department of Health Behavior, Texas A&M University, College Station, TX, United States

**Keywords:** healthy aging, lifestyles and behavior, interventions, technology, environment, implementation science

Consistent with the United Nations Decade of Healthy Aging (1), the Specialty Chief Editors of the Aging and Public Health section of Frontiers in Public Health issued a Research Topic to solicit Expert Opinions on Aging and Public Health. The overall goal was to highlight recent advances in conceptualization, measurement, and intervention strategies to further understanding about the needs of a rapidly aging world and promote healthy aging in diverse settings. This Research Topic addresses the most salient factors that act as facilitators or barriers to healthy aging including: (1) lifestyle and behavior; (2) interventions for healthy aging; (3) technology and innovations; (4) built and social environments; and (5) workforce development for public health and aging. Additionally, it recognizes the importance of precision health, health equity, and implementation science. In total, the Research Topic contains nine expert opinions from *international leaders* whose seminal research and thinking have shaped the way we conceptualize aging in the field of public health.

This Research Topic reflects and reinforces major themes in aging and public health research and practice, such as the importance of interdisciplinary team science research and participatory research approaches (2). As part of a new Australian government research program designed to identify better solutions to major healthcare threats, Thomas et al. conceptualize a transformative model for chronic disease prevention and management that integrates precision medicine, behavior change programs, and digital health solutions. The INTEGRATE model addresses both physical and mental health across the life course. Building on this integration theme, Dubé et al. use social isolation and the aging brain test case to recognize that aging is more than decrements and tout the power of positive attributes (e.g., multi-level resilience). They coined the term “precision convergence” to bridge biology and neuroscience mechanisms with social systems that impact real-world behavior. This integrative approach supports the centrality of health equity as a major goal of aging and public health research and practice. Summarizing lessons learned from a virtually-hosted Health Equity Innovation Summit in personalized medicine, Ory et al. emphasize that new paradigms are needed to ensure the inclusion of underrepresented populations, and especially older adults. Underscoring the importance of community collaborations to achieve equitable access to state-of-the-art treatments, they conclude that for maximal population health, personalized medicine must be re-envisioned to consider the full continuum of care and care partners.

Technology has become ever-abundant in the 21st century and is often viewed as a solution for limited human resources (3). Yet, understanding the true value of technology requires a social technology approach. Kleinman et al. known for their understanding of the intersection of health and culture, posit several social technology tenets for assisting older adults, their families, and communities. These include fostering multidisciplinary collaboration, attending to ethical and humanistic standards, engaging with social service providers, promoting social justice; enhancing social integration through age-friendly and intergenerational programs; and employing best practices. Although technology-based care options for older adults are expanding widely, harmful stereotypes about their technology-related abilities, preferences, and fears may hinder access and engagement in research studies. Clair et al. provide several examples of their community-centered approaches where researchers can learn from their participants and reduce “researcher bias” by employing person-centered research strategies.

Evidence suggests the environment substantially influences lifestyle behaviors and associated health outcomes for older adults (4). Reflecting on the World Health Organization (WHO) International Active Aging Framework (5), Portegijs et al. explicate the advantages of activity-friendly environments for healthy aging, especially when viewing complex dynamic interactions underlying different physical, social, and technological environments. Their commentary amplifies research from a prior Frontiers in Public Health Research Topic on Healthy Aging and the Community Environment (6). Additionally, social environments have received increased attention with the awareness that social isolation and loneliness are major health risk factors akin to previously documented lifestyle behaviors like smoking (7, 8). Smith et al. offer nine actionable societal- and community-level solutions to help communities collaboratively combat social disconnectedness and its deleterious health outcomes. These recommendations reflect a consensus of several national and international working groups to better conceptualize and measure social connectedness.^1^

The Research Topic concludes with two cross-cutting implementation issues. The first is an appreciation of the global health professional shortage, especially for geriatric care, which negatively impacts the quality-of-care older adults receive (9). This is a long-standing problem that is exacerbating with population aging and fallout from the COVID-19 pandemic. In this context, Evashwick muses about strategies for reducing shortages in geriatric care through accrediting entities' greater recognition of, and requirement for, geriatric competencies in all fields and across all levels of training.

The second cross-cutting issue reinforces the importance of dissemination and implementation science (DIS) for understanding the public health impact of programs, policies and practices (10). Estabrooks and Glasgow, founding members of the National Consortium on RE-AIM Planning and Evaluation Framework (www.re-aim-org), reflect on processes involved in developing a DIS research agenda for aging and public health. Drawing on their experiences with the RE-AIM and PRISM frameworks, they stress the importance of recognizing contextual factors and addressing health inequities. This concluding article underscores the evolution of various DIS frameworks and poses key questions about the “what, when, how, and why” of DIS research that will help accelerate the translation of evidence-based interventions for an aging world.

In summary, contributing experts from Australia, North America, Asia, and Europe provide thought-provoking commentary in this Research Topic on major concepts, methods, and interventions to advance research and practice on aging and public health.

## Author contributions

MO: Conceptualization, Writing—original draft. MS: Conceptualization, Writing—review & editing.

## Footnotes

^1^Coalition to End Social Isolation and Loneliness (CESIL). Available online at: https://www.endsocialisolation.org/ (accessed October 14, 2023).
